# Efficiency of graft-transmitted JcFT for floral induction in woody perennial species of the *Jatropha* genus depends on transport distance

**DOI:** 10.1093/treephys/tpab116

**Published:** 2021-09-09

**Authors:** Mingyong Tang, Xue Bai, Jingxian Wang, Tao Chen, Xin Meng, Hongjun Deng, Chaoqiong Li, Zeng-Fu Xu

**Affiliations:** CAS Key Laboratory of Tropical Plant Resources and Sustainable Use, Xishuangbanna Tropical Botanical Garden, The Innovative Academy of Seed Design, Chinese Academy of Sciences, Menglun, Yunnan 666303, China; Center of Economic Botany, Core Botanical Gardens, Chinese Academy of Sciences, Menglun, Mengla, Yunnan 666303, China; CAS Key Laboratory of Tropical Plant Resources and Sustainable Use, Xishuangbanna Tropical Botanical Garden, The Innovative Academy of Seed Design, Chinese Academy of Sciences, Menglun, Yunnan 666303, China; School of Life Sciences, University of Chinese Academy of Sciences, Beijing 100049, China; CAS Key Laboratory of Tropical Plant Resources and Sustainable Use, Xishuangbanna Tropical Botanical Garden, The Innovative Academy of Seed Design, Chinese Academy of Sciences, Menglun, Yunnan 666303, China; School of Life Sciences, University of Science and Technology of China, Hefei 230027, China; CAS Key Laboratory of Tropical Plant Resources and Sustainable Use, Xishuangbanna Tropical Botanical Garden, The Innovative Academy of Seed Design, Chinese Academy of Sciences, Menglun, Yunnan 666303, China; School of Life Sciences, University of Chinese Academy of Sciences, Beijing 100049, China; CAS Key Laboratory of Tropical Plant Resources and Sustainable Use, Xishuangbanna Tropical Botanical Garden, The Innovative Academy of Seed Design, Chinese Academy of Sciences, Menglun, Yunnan 666303, China; CAS Key Laboratory of Tropical Plant Resources and Sustainable Use, Xishuangbanna Tropical Botanical Garden, The Innovative Academy of Seed Design, Chinese Academy of Sciences, Menglun, Yunnan 666303, China; College of Life Science and Agronomy, Zhoukou Normal University, Wenchang Street, Zhoukou, Henan 466001, China; CAS Key Laboratory of Tropical Plant Resources and Sustainable Use, Xishuangbanna Tropical Botanical Garden, The Innovative Academy of Seed Design, Chinese Academy of Sciences, Menglun, Yunnan 666303, China; State Key Laboratory for Conservation and Utilization of Subtropical Agro-Bioresources, College of Forestry, Guangxi University, Guangxi, Nanning 530004, China

**Keywords:** Euphorbiaceae, florigen transport, *FLOWERING LOCUS T*, grafting, *Jatropha curcas*, RNA interference

## Abstract

*FLOWERING LOCUS T* (*FT*) promotes flowering by integrating six genetic pathways. In *Arabidopsis*, the FT protein is transported from leaves to shoot apices and induces flowering. However, contradictory conclusions about floral induction via graft-transmitted FT in trees were reported in previous studies. We obtained extremely early-flowering transgenic woody *Jatropha curcas L.* by overexpression of *J. curcas FT* using *Arabidopsis thaliana SUCROSE TRANSPORTER 2 (SUC2)* promoter (*SUC2*:*JcFT*) and non-flowering transgenic *J. curcas* by RNA interference (RNAi), which were used to investigate the function of graft-transmitted JcFT in floral induction in woody perennials. Scions from five wild-type species of the *Jatropha* genus and from *JcFT-*RNAi transgenic *J. curcas* were grafted onto *SUC2*:*JcFT* rootstocks. Most grafted plants produced flowers in 1–2 months, and the flowering percentage and frequency of various grafted plants decreased with increasing scion length. Consistently, FT protein abundance in scions also decreased with increasing distance from graft junctions to the buds. These findings suggest that FT proteins can be transmitted by grafting and can induce the floral transition in woody perennials, and the efficiency of graft-transmitted JcFT for floral induction depends on the scion length, which may help explain previous seemingly contradictory observations regarding floral induction via graft-transmitted FT in trees.

## Teaser

*Jatropha curcas* florigens (JcFT proteins) can be transmitted by grafting to induce floral transition in woody perennial species of the *Jatropha* genus, and the efficiency of graft-transmitted JcFT for floral induction depends on the transporting distance.

## Introduction

Flowering, which involves a developmental phase change from a vegetative state to a reproductive state, is of fundamental importance to the plant life cycle. In the model species *Arabidopsis thaliana*, six pathways, i.e., the photoperiod, vernalization, plant hormone, autonomous, ambient temperature and age pathways, coordinate flowering time with environmental inputs to optimize plant adaptation and reproductive success (Albani and Coupland 2010). These pathways converge via a small number of key genes recognized as floral integrator genes, such as *FLOWERING LOCUS T* (*FT*), *SUPPRESSOR OF OVEREXPRESSION OF CONSTANS 1* (*SOC1*) and *LEAFY* (*LFY*), to initiate the early stages of flowering (Blázquez and Weigel 2000, Pena et al. 2001, Kobayashi and Weigel 2007, Xu et al. 2012, Khan et al. 2014). Under long days, FT is rhythmically activated in leaves by CONSTANS (CO), a central activator involved in the photoperiod pathway (Kardailsky et al. 1999, An et al. 2004, Valverde et al. 2004). FT proteins antagonize TERMINAL FLOWER 1 (TFL1) proteins during inflorescence development by competition for complex formation with 14-3-3 and Flowering Locus D (FD) proteins (Turck et al. 2008, Kaneko-Suzuki et al. 2018, Zhu et al. 2021). Recently, a bHLH transcription factor MYC3 was found to repress flowering by antagonizing CO to regulate FT expression (Bao et al. 2019).

The florigen FTs are small, globular and phosphatidylethanolamine binding proteins (PEBPs), which play a crucial role in the transition from vegetative growth to flowering and the integration of flowering signals (Putterill and Varkonyi-Gasic 2016). In annuals, FT proteins have been identified as mobile flowering signals that are produced in the leaves and translocated long distances through the phloem to the shoot apex, triggering flower initiation, as shown in tomato and tobacco (Lifschitz et al. 2006), *Arabidopsis* (Huang et al. 2005, Corbesier et al. 2007, Notaguchi et al. 2008, Yoo et al. 2013*a*, 2013*b*), rice (Tamaki et al. 2007) and cucurbits (Lin et al. 2007, Turck et al. 2008, Turnbull 2011, Wigge 2011, Liu et al. 2013, Yoo et al. 2013*a*). Studies in *Arabidopsis* have shown that the long-distance movement of FT proteins from leaves to shoot apices is controlled by several regulators. The FT INTERACTING PROTEIN 1 (FTIP1), an endoplasmic reticulum-localized protein of the multiple C2 domains and transmembrane protein (MCTPs) family, is crucial for the selective transport of FTs from companion cells to sieve elements, thus affecting FT transport to the shoot apical meristem (Liu et al. 2012, 2013). Furthermore, MCTP-SNARE complex-mediated endosomal trafficking is necessary for FT protein export from phloem companion cells to induce flowering (Liu et al. 2019). This process is controlled in a temperature-dependent manner, and the transcriptional inhibition of FT is induced under low temperature to ensure the optimal flowering conditions of plants (Liu et al. 2020). In addition, SODIUM POTASSIUM ROOT DEFECTIVE 1 (NaKR1), a heavy metal-associated (HMA) domain-containing protein, plays an essential role in regulating the long-distance movement of FT. Loss of function of NaKR1 compromises FT transport to shoot apices via sieve elements, resulting in delayed flowering under long-day conditions (Zhu et al. 2016).

With respect to perennials, contradictory observations regarding floral induction via graft-transmitted FT in trees were reported in previous studies. Grafting studies of poplar and apple with rootstocks expressing *FT* transgenes under the control of a heat-shock inducible promoter *GmHsp17.6-L* (Zhang et al. 2010) or *Gmhsp17.5-E* (Wenzel et al. 2013) did not result in precocious flowering of the receptor scions (Zhang et al. 2010, Wenzel et al. 2013). Bull et al. (2017) demonstrated the overexpression of *Arabidopsis FT* under control of a strong constitutive cauliflower mosaic virus (CaMV) 35S RNA (35S) promoter to trigger early flowering in cassava (*Manihot esculenta*), but failed to induce precocious flowering in non-transgenic scions grafted onto the *FT* transgenic rootstocks. Similarly transgenic overexpression of endogenous *MeFT1* driven by the cassava vein mosaic virus (CsVMV) promoter produced early flowering in cassava, but the *MeFT1* transgenic rootstock was also unable to induce flowering in long-term observations of non-transgenic scions (Odipio et al. 2020). However, Ye et al. (2014) showed, by using a weak synthetic *G10-90* promoter, the early-flowering trait of the *JcFT*-overexpressing transgenic *Jatropha curcas* was graft-transmissible. Song et al. (2019) also reported that transgenic 35S:*VcFT* rootstocks promoted the flowering of non-transgenic scions in blueberry. Recently, Soares et al. (2020) observed that transgenic citrange rootstocks expressing the *Citrus clementina FT3* gene under the control of the *A. thaliana* phloem-specific *SUCROSE TRANSPORTER 2* (*SUC2*) promoter induced precocious flowering in non-transgenic sweet orange scions. Although floral promotion via graft transmission of FT has not been well demonstrated in many tree species, *FT*-like genes have been successfully applied to reduce the long juvenile (pre-flowering) phase of several tree species, enabling rapid breeding (Putterill and Varkonyi-Gasic 2016, Sinn et al. 2021).

To further demonstrate the efficiency of graft-transmitted JcFT for floral induction in perennial plants, in this study, we employed the extremely early-flowering transgenic *J. curcas* overexpressing *JcFT* (*SUC2*:*JcFT*; Li et al. 2014) and the non-flowering transgenic *J. curcas* obtained by RNA interference (RNAi), together with five perennial woody species in genus *Jatropha* of the family Euphorbiaceae, including *J. curcas*, *J. gossypifolia* L., *J. integerrima* Jacq., *J. multifida* L. and *J. Podagrica* Hook., which are important sources of lipids and secondary metabolites (Sujatha et al. 2013, Cavalcante et al. 2020). Here, we demonstrate that JcFT proteins can be transmitted by grafting and promote flowering in five woody perennial species of the *Jatropha*. The abundance of JcFT in the buds of the scions decreased with increasing scion length, and thus the efficiency of graft-transmitted JcFT for floral induction depends on scion length. Our findings help explain the previous seemingly contradictory observations regarding floral induction via graft-transmitted FT in trees.

## Materials and methods

### Vector construction and J. curcas transformation

To construct the *JcFT*-RNAi expression vector, the sense and antisense fragments of *JcFT* were amplified using the primers XK220/221 with XhoI and KpnI restriction sites and XK222/223 with XbaI and BamHI restriction sites, respectively. The two fragments were then ligated to a pHANNIBAL vector (Smith et al. 2000, Wesley et al. 2001) in opposing orientations on either side of a PDK intron to produce a single self-complementary hairpin RNA (hpRNA) of *JcFT* driven by the 35S promoter, after which the expression cassette was cut by *Not*I and subsequently ligated into a pJL10 binary expression vector (Li et al. 2017). The expression vector was transformed to *J. curcas* via the *Agrobacterium*-mediated transformation method (Fu et al. 2015). The sequences of primers used were as follows: XK220 (GCTCGAGTTTTGGGCAAGAGATAGT), with an XhoI site; XK221 (AGGTACCAGTGTTGAAATTCTGACG), with a KpnI site; XK222 (GTCTAGATTTTGGGCAAGAGATAGT), with an XbaI site and XK223 (GATCGATAGTGTTGAAATTCTGACG), with a ClaI site.

### Plant materials and growth conditions

Seeds of *J. curcas*, *J. gossypifolia*, *J. integerrima*, *J. multifida* and *J. podagrica* were collected during the summer from the Xishuangbanna Tropical Botanical Garden (XTBG; 21°54′ N, 101°46′ E, 580 m above sea level) of the Chinese Academy of Sciences located in Mengla County, Yunnan Province, Southwest China. All seeds were germinated, after which all the seedlings were grown in the same greenhouse (at 28 °C, 14 h of light and 10 h of darkness). *SUC2*:*JcFT* plants were obtained from our previous study (Li et al. 2014). Since the regenerated *SUC2*:*JcFT* shoots with flower buds did not produce roots in root induction media, we grafted the *SUC2*:*JcFT* shoots onto rootstocks of wild-type (WT) seedlings. Twenty-five lines (T0) of *SUC2*:*JcFT* transgenic plants were obtained from more than 100 grafted shoots. Only the *SUC2*:*JcFT* shoots with both flower buds and leaves survived and grew to maturity on rootstocks of WT plants. We obtained T1 transgenic seeds from *SUC2*:*JcFT* plants by bagging their parents and T1 transgenic seeds from *JcFT*-RNAi by grafting *JcFT*-RNAi scions onto *SUC2*:*JcFT* rootstocks. Mature transgenic *J. curcas* plants were transplanted into the field within the XTBG and grown from 2012 to 2021. All tissues for real-time quantitative reverse transcription polymerase chain reaction (qRT-PCR) and western blots were immediately frozen in liquid N_2_ and stored at −80 °C until use. The phenotypes of the grafted plants were analyzed. For each *Jatropha* genotype, more than 25 plants were used for characterization.

### Plant grafting

Two-month-old *JcFT*-RNAi *J. curcas* plants and five species of wild-type (WT) *Jatropha* (*J. curcas*, *J. gossypifolia*, *J. integerrima*, *J. multifida* and *J. podagrica*) plants were used as scions. Two-month-old WT plants and *SUC2*:*JcFT J. curcas* plants were used as rootstocks. Stem segments (5 cm in length) were grafted onto WT and *SUC2:JcFT* rootstocks via cleft grafting with a double-edged razor blade. The wound was wrapped up in wrapping film, which was subsequently removed 10 days later. The grafted plants were grown under low-light intensity conditions (<100 μmol m^−2^ s^−1^) for the first 10 days, after which they were grown under high-light intensity conditions (325 μmol m^−2^ s^−1^).

**Figure 1.**
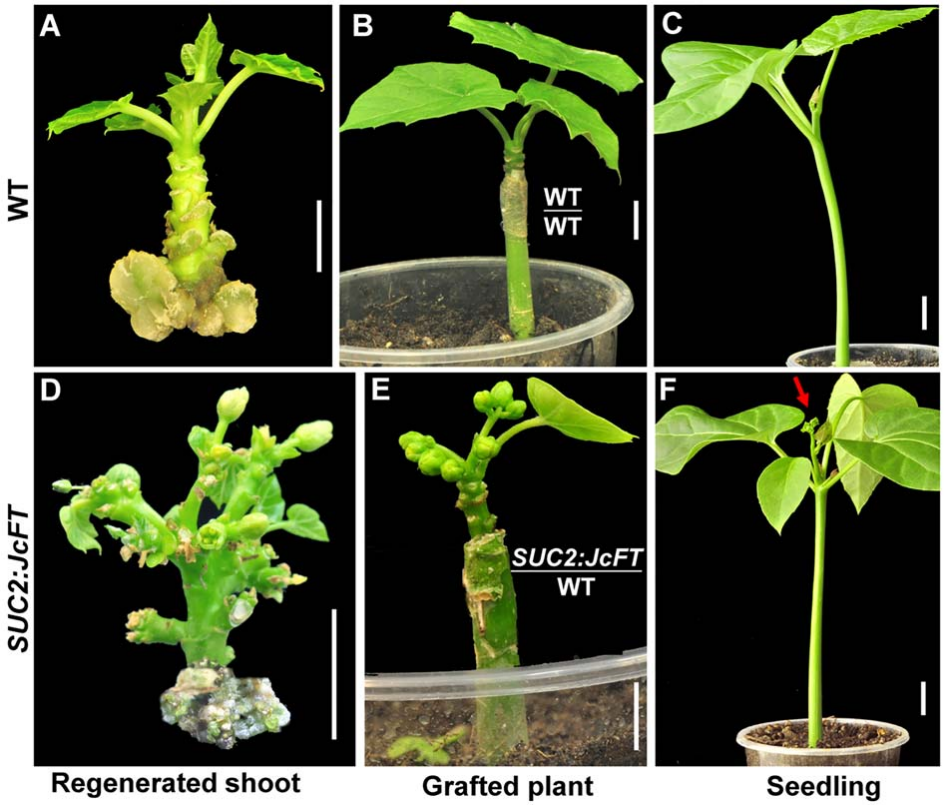
Overexpression of *JcFT* promotes flowering in transgenic *J. curcas*. (A–C) Wild-type (WT) regenerated shoot in vitro (A), WT shoot grafted onto WT rootstock grown for 40 days (B) and 15-day-old seedling (C); (D–F): *SUC2*:*JcFT* transgenicshoot (D), *SUC2*:*JcFT* shoot grafted onto WT rootstock grown for 40 days (E) and 15-day-old T1 *SUC2*:*JcFT* transgenic seedling (F). The red arrow indicates the first inflorescence. Bar =1 cm.

### qRT-PCR analysis

Total RNA was extracted from frozen *Jatropha* tissues as described by Ding et al. (2008). First-strand cDNA was synthesized from 1 μg of total RNA using a PrimeScript^®^ RT Reagent Kit together with gDNA Eraser (Takara, Dalian, China). The cDNA templates were diluted five times with first-strand cDNA using sterilized double-distilled water; qRT-PCR was performed using SYBR^®^ Premix Ex Taq™ II (Takara) on a Roche 480 Real-Time PCR Detection System (Roche Diagnostics, Indianapolis, IN, USA). The primers used for qRT-PCR are listed in Table S1 available as Supplementary data at *Tree Physiology* Online. qRT-PCR was performed on three independent biological replicates, with three technical replicates per sample. The data were analyzed via the 2^−ΔΔCT^ method as described by Livak and Schmittgen (2001). The transcript levels of specific genes were normalized to those of the *Jatropha Actin1* gene.

### Flowering time statistics

The flowering time of the grafted plants grown in the greenhouse was analyzed, and the time from grafting to the first visible inflorescence bud was recorded. The percentage of flowering grafted plants was calculated on the basis of the number of flowering plants and non-flowering plants. The number of inflorescences produced by each grafted plant was recorded and analyzed, and the distance from the junction to the inflorescence buds was also measured. Statistical Package for the Social Science (SPSS) software was used for the data analysis, and SigmaPlot 11.0 software was used for generating graphs.

**Figure 2.**
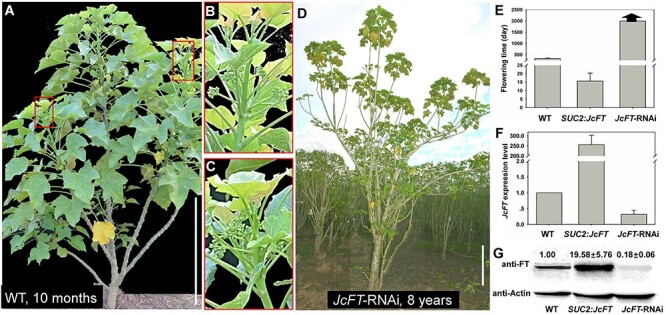
*JcFT*-RNAi transgenic *J. curcas* did not produce flowers. (A) Ten-month-old WT *J. curcas* plant that produced flowers. (B, C) Inflorescences produced from WT plants in (A). (D) Eight-year-old T0 *JcFT*-RNAi transgenic *J. curcas* plant that did not produce flowers. (E) Comparison of flowering time among WT, *SUC2*:*JcFT* and *JcFT*-RNAi plants. The arrows at the top of bar indicate the plants that have not flowered. (F) Analysis of *JcFT* expression levels in WT, *SUC2*:*JcFT* and *JcFT*-RNAi plants by qRT-PCR. RNA was extracted from mature leaves, and the transcript levels were normalized using the *JcACTIN1* gene as a reference. (G) Western blot analysis of JcFT protein abundance in WT, *SUC2*:*JcFT* and *JcFT*-RNAi plants. The values are the means ± standard deviations of three plants per line (three independent experiments). The protein level in the WT was set as the standard, with a value of 1. Scale bars = 50 cm.

### Protein extraction

The shoots (10 cm) from plants of which scions from *J. gossypifolia*, *J. integerrima*, *J. multifida* or *J. podagrica* were grafted onto WT and *SUC2*:*JcFT* seedlings grown in an artificial climate chamber were collected to isolate their total protein. Apical and lateral buds from different scion positions were collected and flash frozen in liquid nitrogen. After grinding, the powder was dissolved in 1 ml of extraction buffer to extract the proteins. The total soluble proteins were isolated using a Plant Total Protein Extraction Kit (No. C500053, Sangon Biotech, Shanghai, China) following the manufacturer’s instructions. The protein concentration was subsequently measured using a Lowry Protein Assay Kit (No. C504031, Sangon Biotech) according to the manufacturer’s instructions.

### SDS-PAGE and western blot analysis

Two hundred micrograms of total protein extracted from *Jatropha* buds and 5x Laemmli buffer (50 mM Tris, 1% SDS, 0.05% bromophenol blue and 10% glycerol [pH 6.8]) were mixed together and incubated at 100 °C for 5 min. The samples were then resolved on 5% spacer and 15% separation discontinuous polyacrylamide SDS gels. Electrophoresis was subsequently conducted at 60 V for 30 min and, followed by 120 V for 60 min. The proteins were transferred onto NC membranes (No. F619511, BBI, Shanghai, China) with a wet electroblotting device (Bio-Rad, Hercules, CA, USA). The transfer conditions included a constant 55 V for 2 h. After transfer of the proteins, the membrane was saturated with 5% skim milk powder (Merck, Darmstadt, Germany) in Tris-buffered saline containing Tween-20 (TBST; 10 mM Tris, 150 mM NaCl, 0.2% Tween-20 [pH 7.5]). Incubation with the primary antibody (polyclonal rabbit anti-FT/Actin antibody, PytoAB, San Jose, CA 95131, USA) was performed overnight at 4 °C; after washing three times in TBST, incubation with the secondary antibody (goat anti-rabbit IgG H&L [HRP]; Dianova, Hamburg, Germany) in TBST was performed at room temperature for 60 min. Bound antibodies were detected with an Immobilon Western Chemiluminescent HRP Detection Kit (Millipore Corporation, Billerica, MA, USA) in conjunction with a Gel Doc™ XR+ system (Bio-Rad).

**Figure 3.**
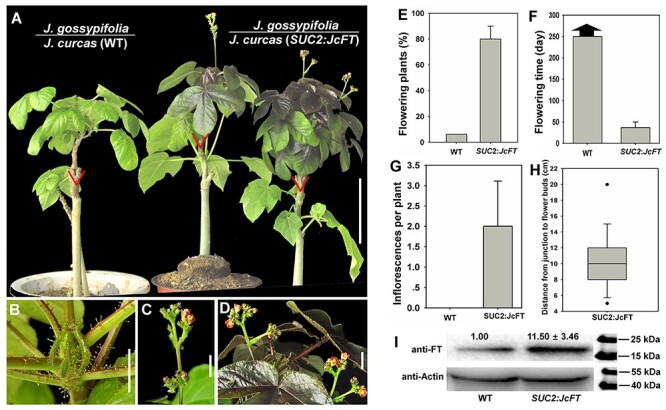
JcFT accelerates *J. gossypifolia* flowering via grafting. (A) Wild-type (WT) *J. gossypifolia* scions grafted onto WT and *SUC2*:*JcFT J. curcas* rootstocks. The triangles indicate the graft junctions. Scale bar = 10 cm. (B) The shoot apex of *J. gossypifolia* grafted onto WT *J. curcas* rootstock. Scale bar = 1 cm. (C, D) Flowers produced on the shoot of *J. gossypifolia* grafted onto *SUC2*:*JcFT J. curcas* rootstock. Scale bar = 1 cm. (E) Comparison of flowering plant percentage of *J. gossypifolia* scions grafted onto WT and *SUC2*:*JcFT J. curcas* rootstocks. (F) Comparison of flowering time of *J. gossypifolia* scions grafted onto WT and *SUC2*:*JcFT J. curcas* rootstocks. (G) Analysis of inflorescences generated on each grafted plant. (H) Analysis of the distance from the graft junction to the flower buds. (I) Western blot analysis of JcFT protein abundance in *J. gossypifolia* scions grafted onto WT and *SUC2*:*JcFT* rootstocks. The values are the means ± standard deviations of three plants per line (three independent experiments). The protein level in the WT was set as the standard, with a value of 1.00. JcFT (20 kDa) and JcActin (45 kDa) were quantified with AtFT and AtActin antibodies. The values are the means ± standard deviations of 15 plants per line (three independent experiments). The asterisks indicate significant differences in comparison with the WT at *P* < 0.05 according to Student’s *t*-test.

**Figure 4.**
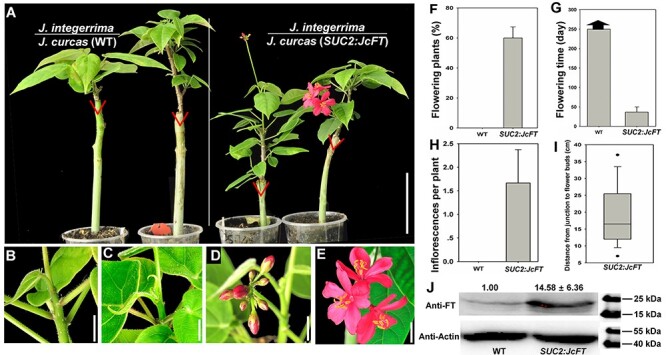
JcFT accelerates *J. integerrima* flowering via grafting. (A) Wild-type (WT) *J. integerrima* scions grafted onto WT (two plants on the left) and *SUC2:JcFT* (two plants on the right) *J. curcas* rootstocks. The triangles indicate the graft junctions. Scale bar = 10 cm. (B, C) The shoot apex of *J. integerrima* grafted onto WT *J. curcas* rootstocks. Scale bar = 1 cm. (D, E) Flowers produced on the shoot of *J. integerrima* grafted onto *SUC2*:*JcFT J. curcas* rootstocks. Scale bar = 1 cm. (F) Comparison of flowering plant percentage of *J. integerrima* scions grafted onto WT and *SUC2*:*JcFT J. curcas* rootstocks. (G) Comparison of flowering time of *J. integerrima* scions grafted onto WT and *SUC2*:*JcFT J. curcas* rootstocks. (H) Analysis of inflorescences generated on each grafted plant. (I) Analysis of the distance from the junction to the flower buds. (J) Western blot analysis of JcFT protein abundance in *J. integerrima* scions grafted onto WT and *SUC2*:*JcFT* rootstocks. The values are the means ± standard deviations of three plants per line (three independent experiments). The protein level in the WT was set as the standard, with a value of 1.00. JcFT (20 kDa) and JcActin (45 kDa) were quantified with AtFT and AtActin antibodies. The values are the means ± standard deviations of 15 plants per line (three independent experiments). The asterisks indicate significant differences in comparison with the WT at *P* < 0.05 according to Student’s *t*-test.

**Figure 5.**
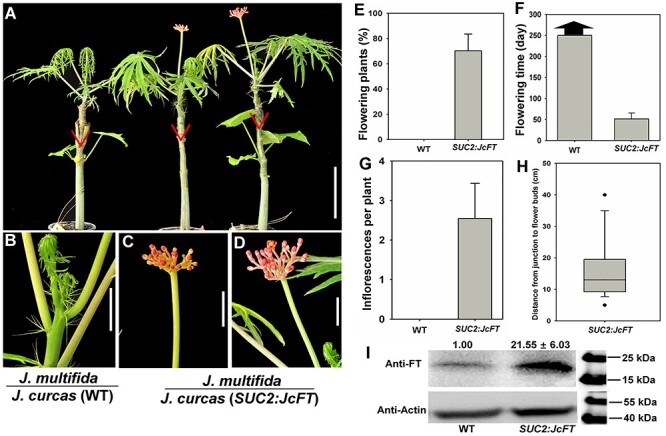
JcFT accelerates *J. multifida* flowering via grafting. (A) Wild-type (WT) *J. multifida* scions grafted onto WT and *SUC2:JcFT J. curcas* rootstocks. The triangles indicate the graft junctions. Scale bar = 10 cm. (B) The shoot apex of *J. multifida* grafted onto WT *J. curcas* rootstock. Scale bar = 1 cm. (C, D) Flowers produced on the shoot of *J. multifida* grafted onto *SUC2*:*JcFT J. curcas* rootstock. Scale bar = 1 cm. (E) Comparison of flowering plant percentage of *J. multifida* scions grafted onto WT and *SUC2*:*JcFT J. curcas* rootstocks. (F) Comparison of flowering time of *J. multifida* scions grafted onto WT and *SUC2*:*JcFT J. curcas* rootstocks. (G) Analysis of inflorescences generated on each grafted plant. (H) Analysis of the distance from the junction to the flower buds. (I) Western blot analysis of JcFT protein abundance in *J. multifida* scions grafted onto WT and *SUC2*:*JcFT* rootstocks. The values are the means ± standard deviations of three plants per line (three independent experiments). The protein level in the WT was set as the standard, with a value of 1.00. JcFT (20 kDa) and JcActin (45 kDa) were quantified with AtFT and AtActin antibodies. The values are the means ± standard deviations of 15 plants per line (three independent experiments). The asterisks indicate significant differences in comparison with the WT at *P* < 0.05 according to Student’s *t*-test.

## Results

### JcFT is essential for floral initiation in J. curcas

To investigate the functions of graft-transmitted JcFT in floral induction in woody perennial species of the *Jatropha* genus, we used transgenic *J. curcas* plants overexpressing *JcFT* driven by the phloem-specific *A. thaliana*  *SUC2* promoter (designated *SUC2*:*JcFT*; Li et al. 2014). Normally, the regenerated shoots in the shoot induction media, the grafted plantlets and seedlings of WT did not produce any flowers (Figure 1A–C). However, *SUC2*:*JcFT* plants flowered extremely early. Flower buds appeared on the regenerated shoots in the shoot induction media (Figure 1D). Since the shoots with flower buds failed to produce roots in root induction media, we grafted the shoots with flower buds onto WT seedling rootstocks and the scions produced flowers continually after grafting (Figure 1E). In addition, the T1 seedlings derived from self-pollinated *SUC2*:*JcFT* transgenic scions produced flowers at 15 days of age (Figure 1F). These results indicate that *JcFT* plays a key role in promoting flowering in *J. curcas*.

To further test whether *JcFT* is essential for *J. curcas* floral induction, we generated *JcFT*-RNAi constructs and transformed them into *J. curcas* cotyledons via *Agrobacterium*-mediated transformation (Fu et al. 2015). We obtained *JcFT*-RNAi transgenic plants, which did not produce any flowers for 8 years, whereas the WT plants usually produced flowers within the first year (Figure 2A–D). The flowering times of the WT and *SUC2*:*JcFT* plants were ~325 and 15 days, respectively (Figure 2E).

These results showed that the *SUC2*:*JcFT* plants flowered extremely early, whereas the *JcFT*-RNAi plants did not flower during an 8-year period. In good agreement with these observations, we measured the mRNA expression level and protein abundance in the transgenic plants by qRT-PCR and western blotting, respectively, and found that both the mRNA and protein levels increased in the *SUC2*:*JcFT* plants but decreased in *JcFT*-RNAi plants. The expression level of *JcFT* in *SUC2*:*JcFT* plants was 250-fold as WT, and the protein abundance was 19.58-fold as WT; the expression level of *JcFT* in *JcFT-RNAi* plants was only 30% as WT, and the protein abundance was 18% as WT (Figure 2F and G).

### Graft-transmitted JcFT promotes flowering in species of the Jatropha genus

Previous research involving ectopic expression in WT and *ft-10* mutant *Arabidopsis* plants indicated that *JcFT* controls flowering (Li et al. 2014). Nevertheless, no research has revealed that the function of *JcFT* is conserved in woody perennial plant species. In this study, we grafted 2-month-old shoots of four other *Jatropha* species, *J. gossypifolia*, *J. integerrima*, *J. multifida* and *J. podagrica*, onto WT and *SUC2*:*JcFT* transgenic *J. curcas* seedlings, respectively. The results showed that the majority of non-transgenic shoots of the four *Jatropha* species produced flowers within a month in an artificial climate chamber after being grafted onto *SUC2*:*JcFT* transgenic seedlings, whereas the other non-transgenic shoots grafted onto WT rootstocks did not produce any flowers under the same conditions (Figures 3A–D, 4A–E and 5A–D, and see Figure S1A available as Supplementary data at *Tree Physiology* Online). The number of inflorescences produced on each chimeric plant ranged from 1.5 to 2.6; although these plants produced more than one inflorescence, they did not bloom continually in the chamber (Figures 3G, 4H and 5G and see Figure S1D available as Supplementary data at *Tree Physiology* Online). We further analyzed the distance from the graft junction to inflorescence buds, and the data showed that the inflorescence buds clustered within a region from 8 to 25 cm from the graft junction (Figures 3H, 4I, 5H and see Figure S1E available as Supplementary data at *Tree Physiology* Online). Finally, using western blots, we measured the JcFT protein abundance in the shoot apices of different *Jatropha* scions grafted onto WT and *SUC2*:*JcFT*plants, and the results showed that the amount of FT in the shoots grafted onto *SUC2*:*JcFT* plants was 11.5–28.5 folds that of the shoots grafted onto the WT plants (Figures 3I, 4J and 5I, and see Figure S1F available as Supplementary data at *Tree Physiology* Online). These results suggest that, by being transported from the rootstock to the scion, JcFT also functions as a flowering accelerator in other woody perennial plant species of the *Jatropha* genus.

### Graft-transmitted JcFT rescues the non-flowering phenotype of JcFT-RNAi transgenic J. curcas

**Figure 6.**
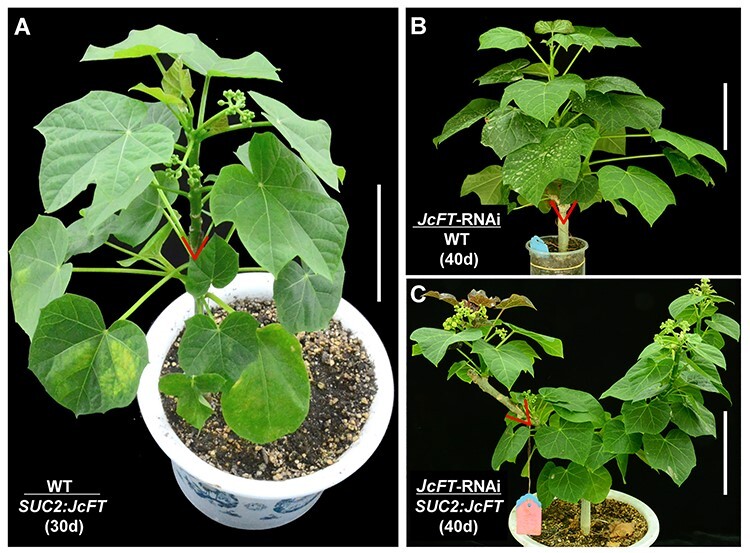
Overexpression of *JcFT* rescued the non-flowering phenotype of *JcFT-*RNAi transgenic *J. curcas.* (A) Wild-type (WT) *J. curcas* scions grafted onto *SUC2*:*JcFT J. curcas* rootstocks. (B) *JcFT*-RNAi *J. curcas* scions grafted onto WT *J. curcas* rootstocks. (C) *JcFT*-RNAi *J. curcas* scions grafted onto *SUC2*:*JcFT J. curcas* rootstocks. The red triangles indicate the graft junctions. Scale bar = 10 cm.

In this study, we found that decreased *JcFT* mRNA and protein levels in *JcFT-*RNAi transgenic *J. curcas* prevented flowering (Figure 2F and G). These *JcFT-*RNAi plants and *SUC2*:*JcFT* plants with phloem-specific *JcFT* expression are therefore ideal materials to determine whether FT can be transported in woody perennials. Therefore, we grafted *35S*:*JcFT*-RNAi transgenic shoots onto *SUC2*:*JcFT* transgenic seedlings. As shown in Figure 6A, WT scions produced flowers ~30 days after being grafted onto *SUC2*:*JcFT* transgenic seedlings. As expected, the *35S*:*JcFT*-RNAi transgenic scions did not produce flowers after being grafted onto WT rootstocks (Figure 6B). However, the *35S*:*JcFT*-RNAi transgenic scions produced flowers ~40 days after grafting onto *SUC2*:*JcFT* transgenic rootstocks (Figure 6C). Hence, through grafting, the *SUC2*:*JcFT* transgenic rootstocks successfully rescued the non-flowering phenotype of *JcFT*-RNAi transgenic *J. curcas* scions, which confirmed that the FT protein but not *FT* mRNA， can be transported from rootstocks to scions by grafting in woody perennials.

### JcFT protein levels decreased with increasing translocation distance

**Figure 7.**
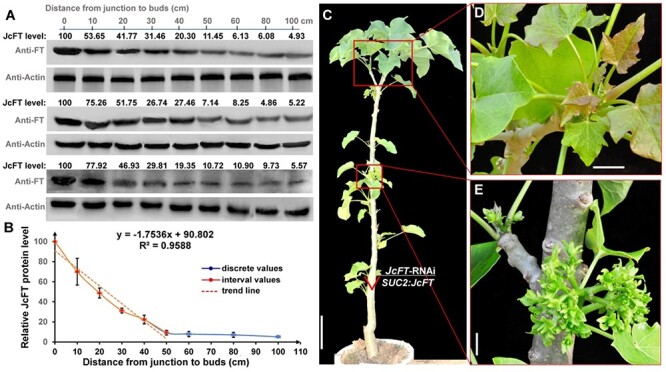
JcFT protein abundance decreased with increasing transport distance. (A) Western blot analysis of JcFT protein abundance in the scions of *JcFT*-RNAi *J. curcas* grafted onto *SUC2*:*JcFT* rootstocks (three independent experiments). The protein level in the rootstock was set as the standard, with a value of 100, and the values of the bands were quantified by Image lab software (Bio-Rad, Hercules, CA, USA.). JcFT (20 kDa) and JcActin (45 kDa) were quantified by AtFT and AtActin antibodies. (B) Relationship between distance from graft junctions to the buds and JcFT protein level. The values were calculated on the basis of three independent experiments, and the numbers are presented as the means ± standard deviations. (C) One-year-old plant derived from a *JcFT*-RNAi scion grafted onto a *SUC2*:*JcFT* rootstock. Scale bar =10 cm. (D) Shoot apex of a *JcFT*-RNAi scion grafted onto the *SUC2*:*JcFT* rootstock in (C). No flowers were produced. Scale bar = 5 cm. (E) Lateral buds of a *JcFT*-RNAi scion grafted onto the *SUC2*:*JcFT* rootstock in (C). Inflorescences were produced. Scale bar = 5 cm. The triangle indicates the graft junction. Scale bar = 10 cm.

After the young shoots of *J. gossypifolia*, *J. integerrima*, *J. multifida* and *J. podagrica* were grafted onto *SUC2*:*JcFT* seedlings, the scions produced flowers in 35.0–57.5 days but did not produce inflorescences continually and the grafted plants produced inflorescences only 1.5–2.5 times in the growth chamber (Figures 3 and 5 and Figure S1 available as Supplementary data at *Tree Physiology* Online). The *SUC2*:*JcFT* rootstocks rescued the *JcFT*-RNAi scions, but no flowers were produced during the second and third years (see Figure S2 available as Supplementary data at *Tree Physiology* Online). These results indicated that the transportability of FT may be limited by travel distance. To confirm this hypothesis, we grafted *JcFT*-RNAi scions of different lengths onto *SUC2*:*JcFT* rootstocks. The results showed that both the percentage of flowering scions and number of inflorescences produced per plant decreased with increasing scion length. When the length of the scion was 40 cm, no flowers were produced (Table 1). Furthermore, FT protein levels in the lateral buds at different positions were detected by western blot using AtFT antibody, together with AtActin antibody used as an internal reference. The results showed that FT abundance decreased with increasing distance from the junction to the buds. The FT abundance in the grafting joint was set as a value of 100, then after 100-cm transporting the FT abundance was decreased to 4.9–5.6 (Figure 7A). In particular, the decreasing tendency was obvious from 0 to 50 cm; however, there was no significant change in the region from 60 to 100 cm (Figure 7A and B). As such, we constructed a fitted curve according to the protein abundance data (inclusive of three independent replications), and an approximation formula (*y* = −1.7536*x* + 90.802) of the matched curve was proposed (Figure 7B). Based on the trend line, it may be difficult to produce flowers when the length of the scions reached 50 cm, which is consistent with our observation that no flowers were produced when the length of the scions was 40 cm (Table 1). Interestingly, some grafted scions produced inflorescences from the lateral buds near the rootstock but did not flower from the apical buds far away from the rootstock (Figure 7C–E). In addition, the grafted trees produced inflorescences in the first year (Figure 6C), but large plants higher than 2 m did not bloom from the grafted scions during the second, third and all subsequent years, whereas flowers and fruits were continually produced from the rootstocks every year (see Figure S2 available as Supplementary data at *Tree Physiology* Online). However, when the length of the scions was shortened to <20 cm, the new regenerated branches produced inflorescences again (see Figure S3 available as Supplementary data at *Tree Physiology* Online).

**Table 1:** Comparison of flowering phenotypes in grafted plants

Rootstocks	Scions	Scion length (cm)	Percentage of flowering plants (%)	Flowering time (day)	Flowering frequency	Distance from graft junction to flower buds (cm)
WT	*JcFT-*RNAi	5	0.00 ± 0.00^e^	>250^a^	0.00 ± 0.00^d^	–
*SUC2*:*JcFT*	WT	5	90.21 ± 7.51^a^	35.00 ± 8.57^c^	2.95 ± 0.73^a^	21.15 ± 9.87^a^
	*JcFT-*RNAi	5	63.74 ± 8.37^b^	53.15 ± 13.90^b^	1.80 ± 0.46^b^	9.08 ± 0.64^c^
		10	46.06 ± 6.35^c^	57.50 ± 14.11^b^	1.11 ± 0.31^c^	19.33 ± 3.33^a^
		20	10.92 ± 2.91^d^	50.76 ± 20.34^b^	1.08 ± 0.14^c^	25.58 ± 7.55^a^
		40	0.00 ± 0.00^e^	>250^a^	0.00 ± 0.00^d^	–

WT and *SUC2*:*JcFT* transgenic plants were used as rootstocks, and WT and *JcFT-*RNAi transgenic shoots were used as scions. The percentage of flowering plants, flowering time and flowering frequency of plants were calculated, and the distance from graft junction to flower buds was analyzed. The length of the rootstocks was 15 cm. The numbers in the table are presented as the means ± standard deviations. The values with different letters are significantly different (*P* < 0.05, Tukey’s test). The experiments were replicated three times, and 20 plants were analyzed in each experiment.

**Figure 8.**
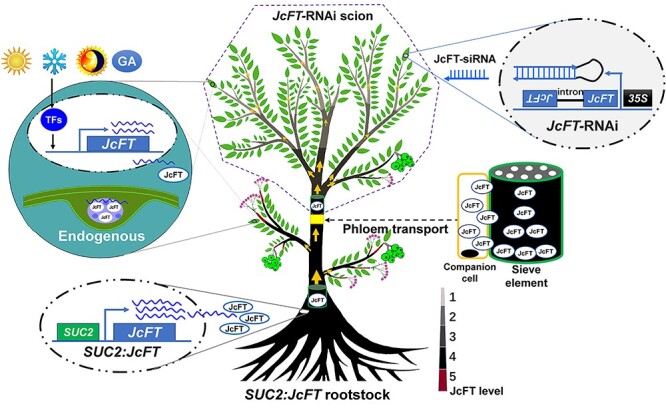
Schematic diagram of *JcFT* transcription, translation and graft transmission from rootstock to scion*. JcFT-*RNAi scions were grafted onto *SUC2*:*JcFT* rootstocks. JcFT protein abundance decreased with increasing distance from the graft junction (yellow rectangle in the middle of the trunk) to the buds. The highest abundance was located in the buds of *SUC2*:*JcFT* rootstocks, which is marked as red, and the varying degrees of blackness in the stem indicate different levels of JcFT protein. The transport directions of the JcFT protein are shown with yellow arrows, and the thickness of the arrows shows the abundance of JcFT protein. 35S, the 35S RNA promoter of cauliflower mosaic virus; GA, gibberellin; *JcFT*, *Jatropha curcas FLOWERING LOCUS T*; OE, overexpression; RNAi, RNA interference; siRNA, small interfering RNA; *SUC2*, the promoter of *Arabidopsis thaliana SUCROSE TRANSPORTER 2*.

## Discussion

### FT plays a conservative role in initiating the vegetative to floral transition via grafting in both perennial and annual plants

In this study, we analyzed the function and transferability of JcFT by constructing *SUC2*:*JcFT* and *JcFT*-RNAi transgenic plants and 13 kinds of grafted plants derived from five species of *Jatropha* genus. The *SUC2*:*JcFT* transgenic plants flowered extremely early (Li et al. 2014, Ye et al. 2014), whereas *JcFT*-RNAi transgenic plants did not bloom (Figure 1D–F and 2D,E). Both mRNA and protein levels consistently increased in the *SUC2*:*JcFT* plants and decreased in the *JcFT*-RNAi plants (Figure 2F and G). These results clearly indicate that the reduced expression of *JcFT* by RNAi prevented the transition from vegetative to reproductive growth. The *JcFT*-RNAi transgenic plants did not bloom, which also indicates that FT plays a vital role in the regulation of flowering in woody perennial plants and that no other genes that are functionally redundant with *JcFT* are present in *J. curcas*.

A large number of grafting experiments performed with many annual species, such as *Arabidopsis* (Corbesier et al. 2007, Lu et al. 2012), tobacco (Freiman et al. 2015), rice (Tamaki et al. 2007, Song et al. 2017), *Cucurbita moschata* (Yoo et al. 2013*a*), cucurbits (Lin et al. 2007), tomato (Lifschitz et al. 2006) and potato (Navarro et al. 2011), have demonstrated that FT proteins are translocated to the shoot apex to initiate floral morphogenesis. Previous studies have shown that overexpression of *FT* orthologs in other woody plant species, such as orange (Endo et al. 2005), poplars (Zhang et al. 2010), plum (Srinivasan et al. 2012), apple (Wenzel et al. 2013) and citrus (Sinn et al. 2021), also results in an early-blooming phenotype. In this study, our results show that JcFT promotes flowering not only in *J. curcas* (Figure 1D–F) but also in other woody perennial plant species, including *J. gossypifolia* (Figure 3C–F), *J. integerrima* (Figure 4D–G), *J. multifida* (Figure 5C–F) and *J. podagrica* (see Figure S1A–C available as Supplementary data at *Tree Physiology* Online). The floral initiation of young WT shoots excised from *J. curcas* (Table 1), *J. gossypifolia* (Figure 3F), *J. integerrima* (Figure 4G), *J. multifida* (Figure 5F) and *J. podagrica* (see Figure S1C available as Supplementary data at *Tree Physiology* Online) was quickly activated after the shoots were grafted onto *SUC2*:*JcFT* rootstocks. Furthermore, through grafting, *SUC2*:*JcFT* transgenic rootstocks successfully rescued the non-flowering phenotype of *JcFT*-RNAi scions (Figure 6C). These results indicate that the mobility of JcFT was extensively existed in perennials of *Jatropha* genus. Taken together, these results clearly demonstrate that the FT protein can be translocated from rootstocks to scions in woody perennials, and also indirectly confirm that the graft-transmitted florigen is FT protein, not FT mRNA, because *JcFT* mRNA would be degraded in *JcFT-*RNAi scions. Overall, we conclude that the transportability and the function in initiating the vegetative to floral transition of florigen FTs are broadly conserved in both annual and perennial plants.

### FT transportability is limited by translocation distance in trees

Although the graft-transportability of FTs has been well demonstrated in annual species, there are some contradictory reports on floral induction via graft-transmitted FTs in trees (Putterill and Varkonyi-Gasic 2016, Zhu et al. 2021). Grafting experiments with rootstocks overexpressing *FT* transgenes revealed no floral induction ability in the receptor scions of poplar (Zhang et al. 2010), apple (Wenzel et al. 2013) and cassava (Bull et al. 2017, Odipio et al. 2020). However, floral induction via graft-transmitted FTs was found in *J. curcas* (Ye et al. 2014), blueberry (Song et al. 2019) and citrus (Soares et al. 2020). To resolve this apparent contradiction, we demonstrate in this study that JcFT transportability was limited by translocation distance from graft junctions to the buds, and JcFT abundance decreased with increasing scion length (Figure 7A and B). The results suggest that FT cannot be transported long distances in woody perennial plants.

The *FT* expression level in the *SUC2*:*JcFT* plants was more than 200-fold higher than that in the WT (Figure 2F), which resulted in that the flowering time of the T1 transgenic seedlings was only 15 days after germination (Figure 1F). Both the percentage of flowering scions and the number of inflorescences produced per grafted plant decreased with increasing scion length (from 5 to 20 cm), and no flowers were produced when the length of the *JcFT*-RNAi scions was 40 cm (Table 1). We speculate that some JcFT might be degraded by serine/cysteine proteases during transportation (Kim et al. 2016, Qin et al. 2017), resulting in a decrease in amount of JcFT transported to buds of scions. Hence, the key to successfully inducing flowering in scions by the graft-transmitted FT from transgenic rootstocks is to shorten the length of the scions. This was further supported by our observation that the new regenerated branches produced inflorescences again when the length of the scions was shortened to <20 cm (see Figure S3 available as Supplementary data at *Tree Physiology* Online). Thus, we propose that the failure of floral induction via graft-transmitted FTs in poplar, apple and cassava (Zhang et al. 2010, Wenzel et al. 2013, Bull et al. 2017, Odipio et al. 2020) may result from a relatively low level of transgene *FT* expression in rootstocks and/or relatively long scions used for grafting experiments. According to the above results, we propose a schematic diagram of *JcFT* transcription, translation and transport from rootstock to scion (Figure 8).

In addition, to obtain *FT* transgenic rootstocks with the ability to induce precocious flowering in non-transgenic scions, the selection of a suitable promoter for driving *FT* transgene expression is crucial. In our previous study (Li et al. 2014), the strong constitutive 35S promoter-driven *JcFT* transgenic shoots with flower buds failed to develop normally, whereas the viable transgenic *Jatropha* shoots were obtained by using the phloem-specific *SUC2* promoter, which were grafted onto rootstocks of WT seedlings. Consistently, Ye et al. (2014) obtained a weak synthetic *G10-90* promoter-driven *JcFT* transgenic *Jatropha* rootstocks, which were capable of promoting flowering in the recipient scions, but failed to generate transgenic *Jatropha* plants overexpressing *JcFT* from a strong constitutive 35S promoter. Recently, Soares et al. (2020) examined four different types of promoters for efficient expression of the *CcFT3* transgene in citrus, including two constitutively expressed promoters (a strong 35S promoter and a weaker *NOPALINE SYNTHASE* [*NOS*] promoter), the phloem-specific *Arabidopsis SUC2* and the heat inducible *A. thaliana HEAT SHOCK PROTEIN 18.2* (*AtHSP18.2*) promoter. Only the phloem-specific *SUC2* promoter-driven *CcFT3* transgenic citrange rootstocks showed normal morphological characteristics, exhibited normal vigor and were capable of inducing precocious flowering in juvenile non-transgenic scions (Soares et al. 2020). Hence, the *Arabidopsis SUC2* promoter could be an excellent phloem specific promoter driving transgene expression of graft-transmitted JcFT for floral induction in woody perennial species.

### Modulation of FT abundance for plant genetic improvement and breeding

For crops, flowering and reproductive cycling are keys for field performance. Modifications of the expression of FT-like genes shifted the proportion of vegetative growth (shoots and leaves) to reproductive growth (flowers, fruits and seeds), providing a range of variation in shoot architecture, plant size, flowering time, fruit set and seed production (Pin and Nilsson 2012, Moraes et al. 2019). This ample variation can be exploited for the genetic studies and breeding of woody perennials. The long juvenile period of woody perennial trees has hindered both genetic studies and traditional breeding. To reduce the generation time in trifoliate orange, a citrus homolog of the *FT* gene was introduced into trifoliate orange, and the transgenic plants showed extremely early flowering and fruiting (Endo et al. 2005), which was used recently in a fast-track breeding system to introduce citrus tristeza virus (CTV) resistance of trifoliate orange into citrus germplasm (Endo et al. 2020). In poplar, overexpression of *Populus trichocarpa FT1* and *FT2* driven by a soybean heat-inducible promoter successfully shortened the generation cycle for breeding (Zhang et al. 2010). A previous study showed that expressing *A. thaliana* or citrus *FT* genes can successfully promote the transition from the vegetative to the reproductive phase in juvenile citrus plants (Velázquez et al. 2016). Recently, Sinn et al. (2021) found moderate expression of a chimeric FT protein resulted in precocious blooming largely without negative effects in edible citrus cultivars, suggesting an additional valuable tool for rapid-cycle citrus breeding. Similarly, constitutive expression of *A. thaliana FT* gene (*AtFT*) in *Eucalyptus* led to very early flowering, which was also considered as an effective means for acceleration of eucalypt tree breeding and genetic studies (Klocko et al. 2016). By taking advantage of the early-continuous flowering transgenic plums overexpressing poplar *FT* (*PtFT1*) (Srinivasan et al. 2012), a novel breeding strategy, termed FasTrack breeding, has been developed to shorten the breeding cycle of plums (Petri et al. 2018).

The early flowering transgenic *J. curcas* plants overexpressing *JcFT* obtained in this study may have potential for reducing the length of the breeding cycle of *Jatropha* species via grafting or crossing (van Nocker and Gardiner 2014). In addition, *JcFT*-RNAi transgenic *J. curcas* has not flowered in 8 years, and the *JcFT*-RNAi plants showed a significant increase in plant height and biomass as compared with WT (Figure 2D and see Figure S4C–F available as Supplementary data at *Tree Physiology* Online). A similar strategy could also be applied to increase the biomass of certain other economically important tree species.

## Supplementary Material

Supplementary_figures_and_table_tpab116

## Data Availability

No new sequence data were published in the present paper. Sequence data included in our manuscript can be obtained from the publicly available genome of *Jatropha curcas* (https://www.ncbi.nlm.nih.gov/bioproject/38697) under the following accession numbers: *JcFT* (NP_001295681), *JcACTIN1* (NM_112764).
