# Supplementary material for: Efficiency of graft-transmitted JcFT for floral induction in woody perennial species of the Jatropha genus depends on transport distance
Source: Tree Physiol. 2021 Sep 9;42(1):189–201. doi: 10.1093/treephys/tpab116 (PMC8755054; doi:10.1093/treephys/tpab116)
Supplement: Supplementary_figures_and_table_tpab116 [file supplementary_figures_and_table_tpab116.pdf]

Efficiency of graft-transmitted JcFT for floral induction in woody perennial species of the *Jatropha* genus depends on scion length

Mingyong Tang[1,3,#,*], Xue Bai[1,2,#], Jingxian Wang[1,5], Tao Chen[1,2], Xin Meng[1], Hongjun Deng[1], Chaoqiong Li[6], Zeng-Fu Xu[1,4,*]

[1] CAS Key Laboratory of Tropical Plant Resources and Sustainable Use, Xishuangbanna Tropical Botanical Garden, The Innovative Academy of Seed Design, Chinese Academy of Sciences, Menglun, Yunnan 666303, China

[2] School of Life Sciences, University of Chinese Academy of Sciences, Beijing 100049, China

[3] Center of Economic Botany, Core Botanical Gardens, Chinese Academy of Sciences, Menglun, Mengla 666303, China

[4] State Key Laboratory for Conservation and Utilization of Subtropical Agro-Bioresources, College of Forestry, Guangxi University, Nanning 530004, Guangxi, China

[5] School of Life Sciences, University of Science and Technology of China, Hefei 230027, China;

[6] College of Life Science and Agronomy, Zhoukou Normal University, Zhoukou, Wenchang Street, Henan, 466001, China.

[*] Correspondence: Mingyong Tang (tangmingyong@xtbg.ac.cn), Zeng-Fu Xu (zfxu@gxu.edu.cn; Tel: +86-691-8713061; Fax: +86-691-8715070)

[#]Mingyong Tang and Xue Bai have contributed equally to this work.

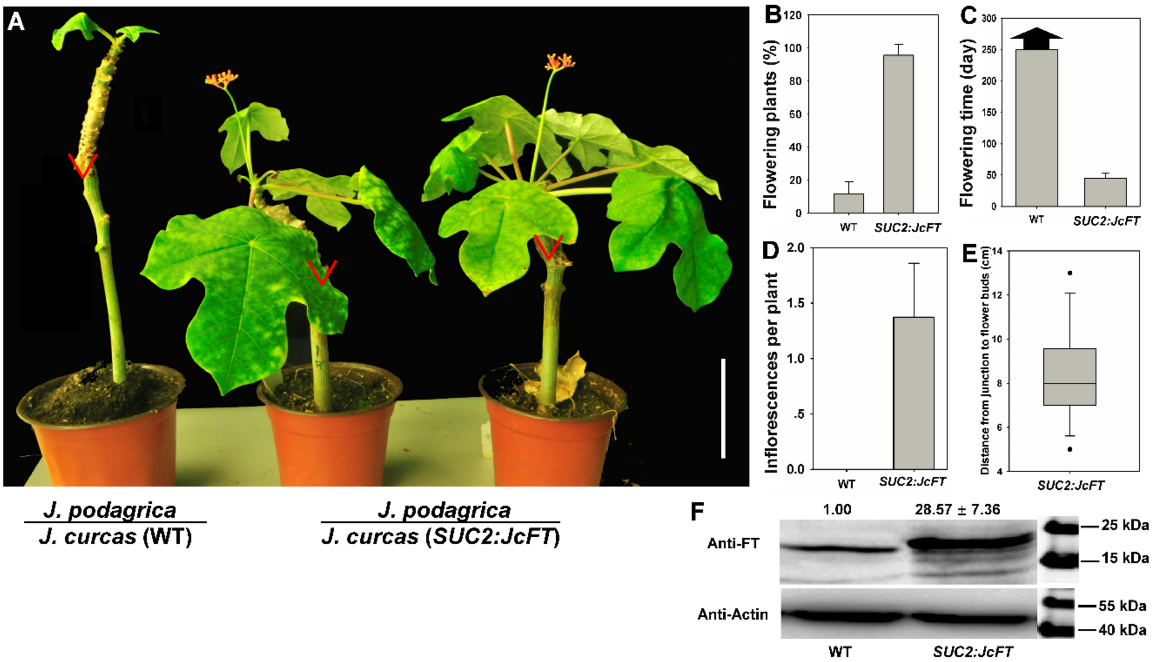

Figure S1. JcFT accelerates *J. podagrica* flowering via grafting.

(A) WT *J. podagrica* scions grafted onto WT and *SUC2:JcFT J. curcas* rootstocks. The triangles indicate the graft junctions. Scale bar = 10 cm; (B) Comparison of flowering plant percentage of *J. podagrica* scions grafted onto WT and *SUC2:JcFT J. curcas* rootstocks; (C) Comparison of flowering time of *J. podagrica* scions grafted onto WT and *SUC2:JcFT J. curcas* rootstocks; (D) Analysis of the number of inflorescences generated on each grafted plant; (E) Analysis of the distance from the graft junction to the flower buds; (F) Western blot analysis of JcFT protein abundance of *J. podagrica* scions grafted onto WT and *SUC2:JcFT* plants. The values are the means ± SDs of 3 plants per line (three independent experiments). The protein level in the WT was set as the standard, with a value of 1.00. FT (20 kDa) and Actin (45 kDa) were quantified via AtFT and AtActin antibodies. The values are the means ± SDs of 15 plants per line (three independent experiments). The asterisks indicate significant differences in comparison with the WT at $P < 0.05$ according to Student's t-test.

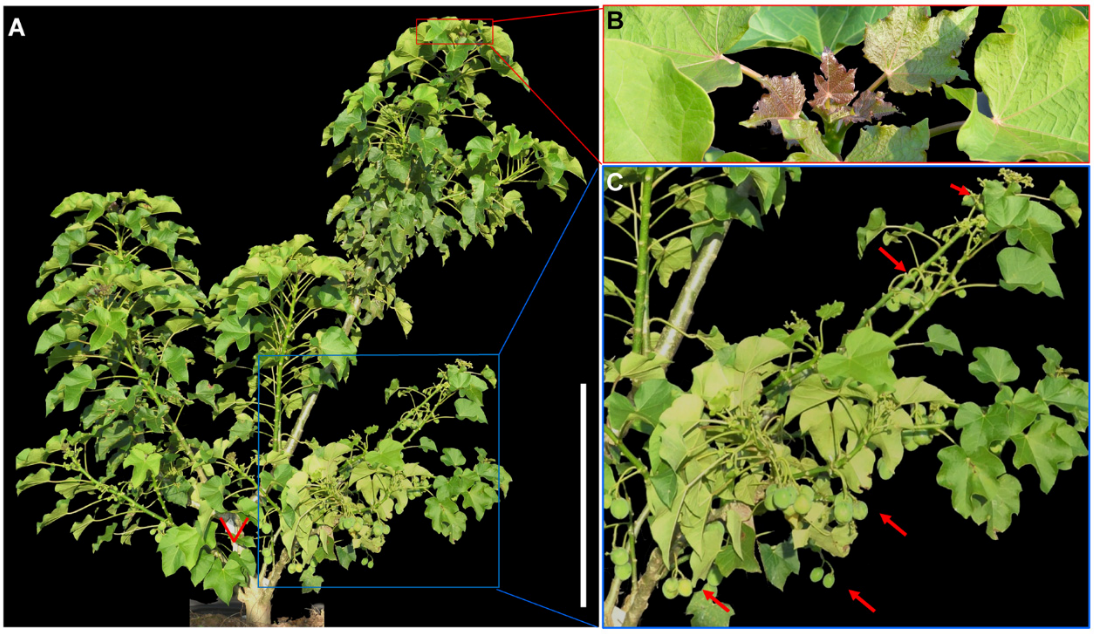

Figure S2. ***JcFT*-RNAi *J. curcas* scions did not produce flowers after being grafted onto *SUC2:JcFT* rootstocks during the second, third or fourth year.**

(A) *JcFT*-RNAi *J. curcas* scions grafted onto WT and *SUC2:JcFT J. curcas* rootstocks. The triangles indicate the graft junctions. Scale bar = 1 m. (B) No flowers were produced in the apical buds of the *JcFT*-RNAi scions; (C) Flowers and fruits were continuously produced on the *SUC2:JcFT* rootstocks.

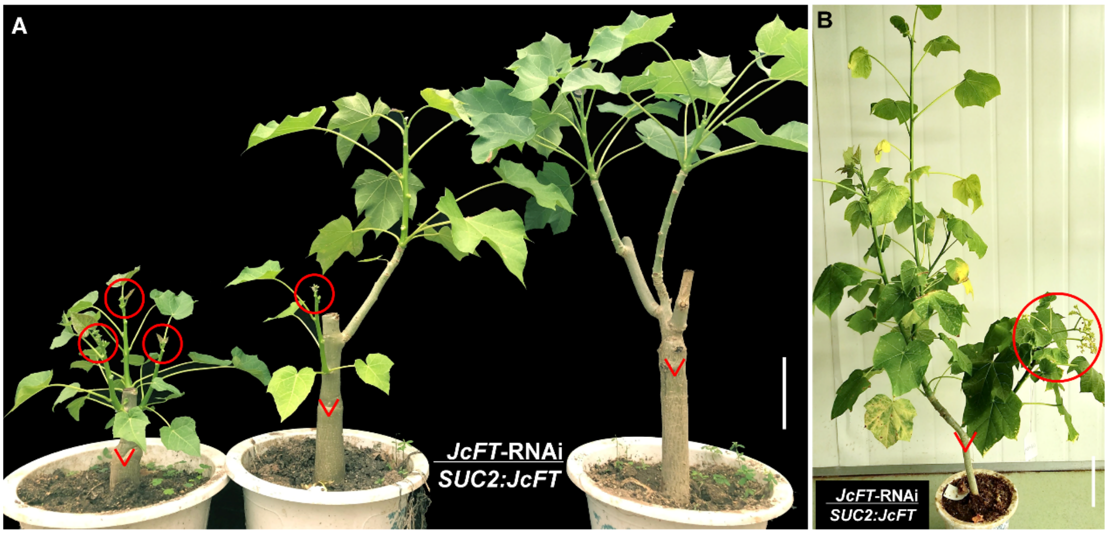

Figure S3. The inflorescences were produced again, one month after the *JcFT*-RNAi scions were shortened in *SUC2:JcFT J. curcas* rootstocks.

(A) The shorter regenerated branches on the scions (left and middle plants) produced inflorescences again, but the longer branches (middle and right plants) did not. (B) The shortened branch (right) on the scion produced inflorescences, whereas the longer branches (left) did not. Scale bar = 10 cm. Red arrows indicate the graft junction, red cycles indicate the inflorescences.

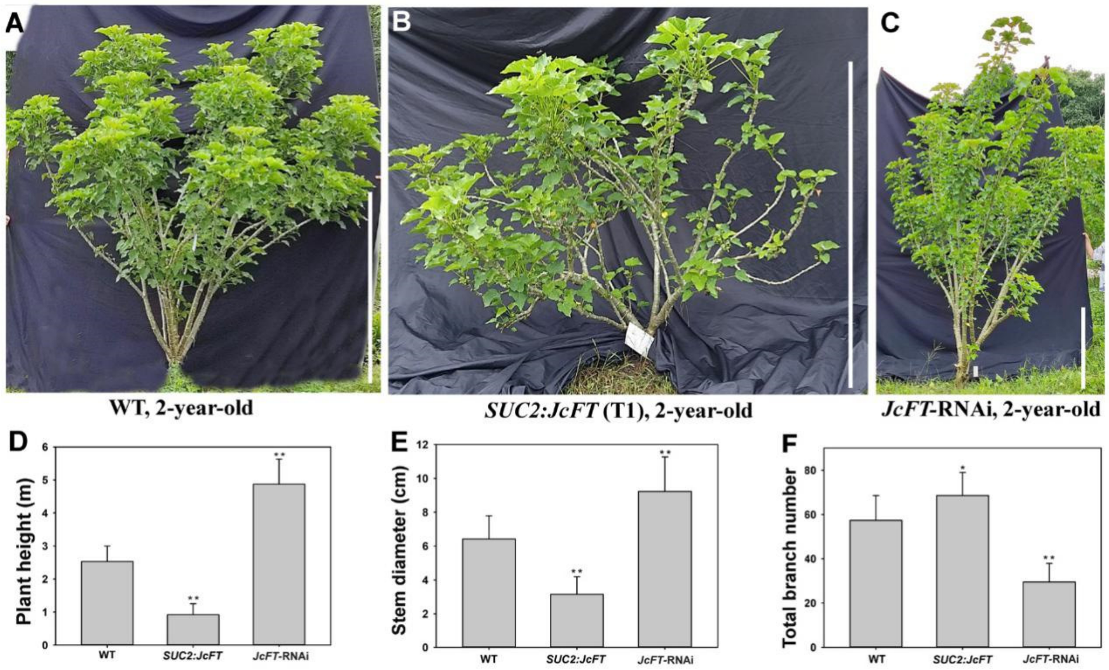

Figure S4. Comparison of tree architecture of WT, *SUC2:JcFT*, and *JcFT*-RNAi plants.

(A-C) Two-year-old WT, *SUC2:JcFT*, and *JcFT*-RNAi plants grown in the field. Bars = 1 m; (D-F) Quantification of height, stem diameter, and branching phenotypes of WT, *SUC2:JcFT*, and *JcFT*-RNAi plants. The values are means ± standard deviations. * Statistically different from the WT at the 5% level, ** Statistically different from the WT at the 1% level. The error bars indicate the standard deviations for 15 plants.

Table S1: List of primers used in this study. All sequences are listed in the 5′ to 3′ direction.

Gene	Primer name	Sequence 5′ -3′
35S promoter	XT126 F	CACTATCCTTCGCAAGACCC
JcFT	XD626 F	TCGTGTGATTGGGGATGTTTTA
identification	XD627 R	TGGTGGATACACGGTCTGCCTT
JcFT	XK686 F	ATGCCTAGGGATCAATTTAGGGACC
qRT-PCR	XK687 R	CCCTAGGTTGGTTGACAACTTGAGA
Jcactin1	XK191 F	CTCCTCTCAACCCCAAAGCCAA
qRT-PCR	XK192 R	CACCAGAATCCAGCACGATACCA
JcFT-RNAi	XK220 F	GCTCGAGTTTTGGGCAAGAGATAGT
construction	XK221 R	AGGTACCAGTGTTGAAATTCTGACG
JcFT-RNAi	XK222 F	GTCTAGATTTTGGGCAAGAGATAGT
construction	XK223 R	GATCGATAGTGTTGAAATTCTGACG
